# Supplementary material for: A comprehensive generalization of the Friendship Paradox to weights and attributes
Source: Sci Rep. 2024 Jun 14;14:13730. doi: 10.1038/s41598-024-63167-9 (PMC11178832; doi:10.1038/s41598-024-63167-9)
Supplement: Supplementary file 1 — Supplementary Information. [file 41598_2024_63167_MOESM1_ESM.pdf]

A comprehensive generalization of the Friendship Paradox to weights and attributes

Anna Evtushenko,[1*] Jon Kleinberg[1,2]

[1]Department of Information Science, Cornell University, Ithaca NY, USA
[2]Department of Computer Science, Cornell University, Ithaca NY, USA

*anna@infosci.cornell.edu

Supplementary Information

Contents

1 An example of a graph that is "weighted-regular" but not regular **3**

2 Directed Friendship Paradox fails for very simple cases **3**

3 Attribute-based Friendship Paradox for unweighted graphs **5**

3.1 list version: LAFP 6

3.1.1 formal definition of the LAFP gap 6

3.1.2 $g_{LAFP} = 0$ for regular graphs and for graphs with no attribute variation 7

3.1.3 the sign of $r_{d,a}$ determines the sign of g_{LAFP} for other graphs 7

3.2 singular version: SAFP 8

3.2.1 formal definition of the SAFP gap 8

3.2.2 $g_{SAFP} = 0$ for regular graphs and for graphs with no attribute variation 9

3.2.3 the sign of $r_{\delta,a}$ determines the sign of g_{SAFP} for other graphs 10

4 Example WAFP failure when degrees are used as attributes 11

5 If all γ_i are equal in a weighted connected graph, it is a weighted-regular graph 12

6 If all δ_i are equal in an unweighted connected graph, it is a regular graph 14

7 8 gaps for one network: a detailed argument 15

7.1 Friendship Paradox 16

7.2 Attribute-based Friendship Paradox 17

7.3 Weighted Friendship Paradox 19

7.4 Weighted Attribute-based Friendship Paradox (EFP) 20

8 Standard deviations for the correlations in the simulation 22

9 An image of the altered example network discussed in the Data section of the main text 23

10 Plots for the Facebook100 configuration model results 23

1 An example of a graph that is “weighted-regular” but not regular

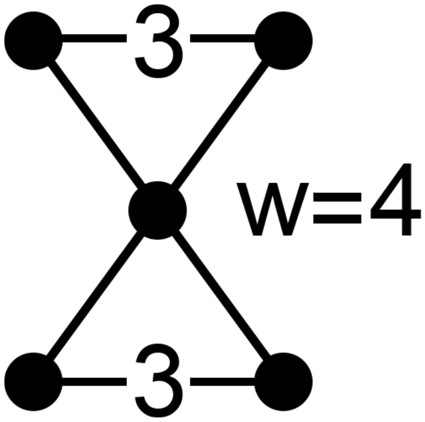

Figure S1: This is a weighted undirected graph on 5 nodes. The weight of each edge, when not 1, is listed on the edge. Each node’s weighted degree is 4 (denoted w in the image), but the degrees of peripheral nodes are 2 and the degree of the central node is 4.

2 Directed Friendship Paradox fails for very simple cases

In this section, we will look at various ways of defining a “Directed Friendship Paradox” and find very simple examples of graphs for which each version of such a paradox fails. This will help us see why directed graphs are difficult to study in the same framework as undirected graphs, and so we should consider undirected graphs only, as we do in the main text of this paper.

To define a version of the paradox for a directed graph, we need to choose what to consider first-order attributes, which edges of node i—their in- or out-edges—to use for considering someone i’s friend for the purposes of computing i’s second-order attribute, and whether to look at list or singular second-order attributes.

To start, let’s consider using out-degrees as first-order attributes. Also, if there is an edge

(i, j), we will say that i considers j their friend. (j would consider i their friend only if there is an edge (j, i)). Thus, i's attribute (out-degree) would be equal to their number of friends. Now, we can calculate the list and the singular gaps for the graph in Figure S2a). There, $a_A = 2, a_B = 1, a_C = 1$. The list gap, then, is equal to $\frac{(1+1)+1+1}{4} - \frac{2+1+1}{3} = -\frac{1}{3}$. The singular gap is $\frac{\frac{1+1}{2}+\frac{1}{1}+\frac{1}{1}}{3} - \frac{2+1+1}{3} = -\frac{1}{3}$. Both are negative.

We can also take in-degrees to be attributes and, separately, say that i would consider j their friend if there is an edge from j to i. In this case, i's attribute (in-degree) is still equal to their number of friends. Then we can fail the list and singular versions of this paradox with an example in Figure S2b). There, we have flipped the direction of all edges in Figure S2a), and the list and the singular gaps are the same as for Figure S2a), and negative.

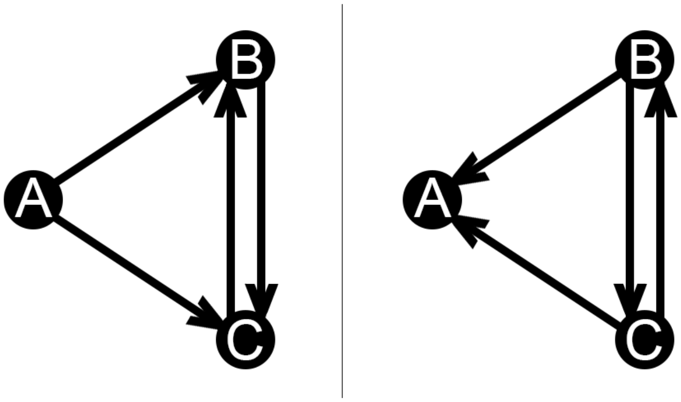

Figure S2: *a)* For this example, the list and the singular versions of the Directed Friendship Paradox fail if we use out-degrees as attributes and say i considers j their friend if there is an edge (i, j). *b)* Reversing the direction of all edges, we get an example for which the list and the singular versions of the paradox fail if we use in-degrees as attributes and say i considers j their friend if there is an edge (j, i).

Finally, it is useful to consider a less-intuitive option: using out-degrees as attributes but saying i considers j a friend if there is an edge (j, i). This is less intuitive because here, a node's attribute is not necessarily equal to their number of friends. And now, it may be possible for a node with many out-links to technically have no friends, like node A in Figure S2a). This

means that for this graph, we can't calculate the singular gap because A's second-order attribute is undefined. While for undirected graphs, it is easy to spot and remove isolates, it is more difficult to remove "bad" nodes from directed graphs as evidenced by this example. The list gap for this version of the paradox and the graph in Figure S2a) is equal to $\frac{()+1+1}{2} - \frac{2+1+1}{3} = -\frac{1}{3}$ which is negative.

The final version to consider is using in-degrees as attributes but saying i considers j a friend if there is an edge (i, j). It is similar to the previous version in that a node's attribute is not necessarily equal to their number of friends. It is also possible to have a node with in-links but no friends, like node A in Figure S2b). This means we can't define the singular gap for this graph and this way of aggregating things. The list gap for this version of the paradox and the graph in Figure S2b) is equal to $\frac{()+1+1}{2} - \frac{2+1+1}{3} = -\frac{1}{3}$ which is negative.

It is possible to come up with specific graphs for which we can define the singular gaps using these non-intuitive ways of aggregating things, and those gaps are negative, but we don't do that due to the issues that these two versions come with.

Overall, directed graphs are different from undirected graphs due to their inherent asymmetry. This leads to difficulties in defining the most basic versions of the paradox in the first place, as well as to gaps being negative in very simple instances. Having examined this, we now go back to considering undirected graphs only.

3 Attribute-based Friendship Paradox for unweighted graphs

While the gap formulas for AFP can be derived from those for WAFP by taking every edge weight to be 1 and replacing any w with d ($w_i = d_i$ for unweighted graphs), we will work them out from scratch here. We will also look at the specific correlation-based rules that determine whether the list version and the singular version fail.

Attribute-based Friendship Paradox is an extension of the original Friendship Paradox that

takes advantage of the fact that any number, not just node's degree, can be averaged over to get second-order values. We call that new number an *attribute*, and the list of attributes an *attribute sequence* a. The attributes are indexed the same way as the degrees so that we can precisely define a *degree-attribute correlation*. Note though that if x consists of all the same values, the correlation between x and y ($r_{x,y}$) is undefined, so we need to look at the case where all degrees are equal and the case where all attributes are equal—meaning where the degree-attribute correlation is undefined—separately. Note that since we are starting from the original FP, we are looking at unweighted graphs.

3.1 list version: LAFP

3.1.1 formal definition of the LAFP gap

List second-order attribute of node i is the list of attributes of i's friends. Mean second-order attribute (list version) is the mean of the concatenation of all such lists for nodes in a graph. The "gap" is the difference between the mean second-order attribute (list version) and the mean attribute in a graph. That is expressed as:

$$g_{LAFP} = \frac{\sum_{i=1}^{n} \sum_{j \in N(i)} a_j}{\sum_{i=1}^{n} d_i} - \frac{1}{n} \sum_{i=1}^{n} a_i \tag{S1}$$

In the expression above, nodes indexed i are "seeds" taking stock of their friends' degrees. We can take a different approach and instead see how many times a friend's degree is featured in a seed's list second-order attribute. A friend with degree d_j (and attribute a_j) is featured in d_j seeds' calculations. The gap can thus be rewritten as:

$$g_{LAFP} = \frac{\sum_{j=1}^{n} a_j d_j}{\sum_{i=1}^{n} d_i} - \frac{1}{n} \sum_{i=1}^{n} a_i$$

Changing j to i and finding a common denominator, this is modified to:

$$g_{LAFP} = \frac{\sum_{i=1}^{n} a_i d_i - \frac{1}{n}\left(\sum_{i=1}^{n} a_i\right)\left(\sum_{i=1}^{n} d_i\right)}{\sum_{i=1}^{n} d_i}$$

We will say that LAFP fails if g_{LAFP} is negative and holds otherwise.

3.1.2 $g_{LAFP} = 0$ for regular graphs and for graphs with no attribute variation

First note that the gap is 0 if the degree sequence or the attribute sequence is constant. This is clear from a direct application of Chebyshev's sum inequality described in the main text, since we can now reindex nodes such that both the degree sequence and the attribute sequence are non-increasing. Now we can further assume that both degrees and attributes have variation, meaning the degree-attribute correlation $r_{d,a}$ is defined.

3.1.3 the sign of $r_{d,a}$ determines the sign of g_{LAFP} for other graphs

Note that if we subtract a constant c from each node's attribute, the gap doesn't change, since each element in the second-order list and the first-order list drops by c, and so the difference between the second-order mean and the first-order mean stays the same. Let's subtract the mean of the attribute sequence from each attribute. Now the attribute sequence mean, denoted $\overline{a}$, is 0, and the gap didn't change.

Taking $\overline{a} = 0$, our gap formula becomes:

$$g_{LAFP} = \frac{1}{\sum_{i=1}^{n} d_i} \sum_{i=1}^{n} d_i a_i$$

with a positive coefficient $\frac{1}{\sum_{i=1}^{n} d_i}$.

The degree-attribute correlation $r_{d,a}$, given $\overline{a} = 0$, is:

$$r_{d,a}(\overline{a}=0) = \frac{\sum_i a_i(d_i - \overline{d})}{\sqrt{\sum_i a_i^2 \sum_i (d_i - \overline{d})^2}}$$

with a positive denominator.

The sign of the correlation is thus determined by the sign of the numerator:

$$\sum_i a_i(d_i - \overline{d})$$

From here, we get

$$\sum_i a_i d_i - \sum_i a_i \overline{d} = \sum_i a_i d_i - \overline{d} \sum_i a_i = \sum_i a_i d_i - \overline{d}0 = \sum_i d_i a_i$$

So, the sign of $r_{d,a}$ is equal to the sign of $\sum_i d_i a_i$. Since g_{LAFP} is $\sum_i d_i a_i$ multiplied by a positive number, the sign of $r_{d,a}$ is equal to the sign of g_{LAFP}.

Recall that LAFP fails if g_{LAFP} is negative and holds otherwise. LAFP holds in cases when $r_{d,a}$ is undefined (when the degrees are all the same and/or the attributes are all the same). If $r_{d,a}$ is defined, LAFP fails if $r_{d,a} < 0$ and holds otherwise. Note that the gap is 0 when $r_{d,a} = 0$, but that does not make a statement about a graph's regularity like it did for LFP and SFP.

LAFP is explored in more detail in Eom et al. (see citation in the main text). In particular, they were the first to show that the sign of $r_{d,a}$ determines the sign of the LAFP gap.

3.2 singular version: SAFP

3.2.1 formal definition of the SAFP gap

Singular second-order attribute of node i is the mean of the attributes of i's friends. Mean second-order attribute (singular version) is the mean of the singular second-order attributes for nodes in a graph. The "gap" is the difference between the mean second-order attribute (singular version) and the mean attribute in a graph. That is expressed as:

$$g_{SAFP} = \frac{1}{n} \sum_{i=1}^{n} \left(\frac{1}{d_i} \sum_{j \in N(i)} a_j \right) - \frac{1}{n} \sum_{i=1}^{n} a_i \tag{S2}$$

In the first term of equation S2, index i refers to nodes (*seeds*) and index j to i's friends, and we look at the seeds' calculations of their second-order attributes. But we can also do the opposite and see how each node j's attribute features in its friends' second-order values. j's coefficient in the second-order mean is equal to $\frac{1}{n}$ times the sum of j's friends' reciprocal degrees:

$$g_{SAFP} = \frac{1}{n}\sum_{j=1}^{n} a_j \left(\sum_{k\in N(j)} \frac{1}{d_k}\right) - \frac{1}{n}\sum_{j=1}^{n} a_j$$

We call $\sum_{k\in N(j)} \frac{1}{d_k}$, the sum of j's friends' reciprocal degrees, δ_j (delta), and could also write:

$$g_{SAFP} = \frac{1}{n}\sum_{j=1}^{n} \delta_j a_j - \frac{1}{n}\sum_{j=1}^{n} a_j \quad \text{(S3)}$$

We will say that SAFP fails if g_{SAFP} is negative and holds otherwise.

3.2.2 $g_{SAFP} = 0$ for regular graphs and for graphs with no attribute variation

If all degrees are the same and equal to d, each δ_j is equal to $\sum_{k\in N(j)} \frac{1}{d_k} = \sum_{k\in N(j)} \frac{1}{d} = d_j \frac{1}{d} = \frac{d}{d} = 1$. Looking at Equation S3, this makes the gap 0.

If all attributes the same and equal to a, using Equation S3, we have $g_{SAFP} = \frac{1}{n}\sum_{j=1}^{n} \delta_j a - \frac{1}{n}\sum_{j=1}^{n} a = \frac{1}{n} a(\sum_{j=1}^{n} \delta_j - n)$. The sign of the gap is equal to the sign of $\sum_{j=1}^{n} \delta_j - n$. The sum of deltas $\sum_{j=1}^{n} \delta_j$ is equal to $\sum_{j=1}^{n} \sum_{k\in N(j)} \frac{1}{d_k}$. In this sum of sums, the term $\frac{1}{d_k}$ is present d_k times, since each of k's friends uses that term in their calculation of delta, and k has d_k friends. Since this argument applies to every node's reciprocal degree, the sum of all deltas is equal to $\sum_{j=1}^{n} d_j \frac{1}{d_j} = \sum_{j=1}^{n} 1 = n$. Thus $\sum_{j=1}^{n} \delta_j - n$ is equal to 0, the sign of the SAFP gap is "none" and the gap itself is 0.

Note also that if a graph is not regular (a case we just looked at), we have variation in deltas. We showed this in a previous paper which was focused on SAFP, but provide the proof again in Section 6 of this document. (Note also that the proof about gammas from Section 5 of this document, which we refer to in the main text, reduces to the proof for deltas if we take all edge

weights to be 1, but again, we want to be explicit and work out the proof for deltas separately.) Once we have established that there is variation in deltas, we can further assume that $r_{\delta,a}$ is defined.

3.2.3 the sign of $r_{\delta,a}$ determines the sign of g_{SAFP} for other graphs

We saw before that subtracting the mean of the attribute sequence from each attribute makes the new attribute mean 0 and doesn't change the gap. Taking $\overline{a} = 0$, our gap formula becomes:

$$g_{SAFP} = \frac{1}{n} \sum_{i=1}^{n} \delta_i a_i$$

with a positive coefficient $\frac{1}{n}$.

The correlation $r_{\delta,a}$, given $\overline{a} = 0$, is:

$$r_{\delta,a}(\overline{a} = 0) = \frac{\sum_i a_i(\delta_i - \overline{\delta})}{\sqrt{\sum_i a_i^2 \sum_i (\delta_i - \overline{\delta})^2}}$$

with a positive denominator.

The sign of the correlation is thus determined by the sign of the numerator:

$$\sum_i a_i(\delta_i - \overline{\delta})$$

From here, we get

$$\sum_i a_i\delta_i - \sum_i a_i\overline{\delta} = \sum_i a_i\delta_i - \overline{\delta}\sum_i a_i = \sum_i a_i\delta_i - \overline{\delta}0 = \sum_i \delta_i a_i$$

So, the sign of $r_{\delta,a}$ is equal to the sign of $\sum_i \delta_i a_i$. Since g_{SAFP} is $\sum_i \delta_i a_i$ multiplied by a positive number, the sign of $r_{\delta,a}$ is equal to the sign of g_{SAFP}.

Recall that SAFP fails if g_{SAFP} is negative and holds otherwise. SAFP holds in cases when $r_{\delta,a}$ is undefined (when the degrees are all the same and/or the attributes are all the same). If $r_{\delta,a}$ is defined, SAFP fails if $r_{\delta,a} < 0$ and holds otherwise. Note that the gap is 0 when $r_{\delta,a} = 0$, but that does not make a statement about a graph's regularity like it did for LFP and SFP.

4 Example WAFP failure when degrees are used as attributes

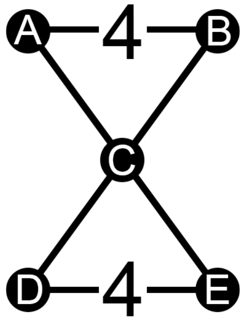

Figure S3: A network on 5 nodes labeled A through E. The weight of each edge, when not 1, is listed on the edge. Node degrees are used as attributes for LWAFP and SWAFP, with edge weights taken into account in second-order calculations.

In the graph above, $w_A = w_B = w_D = w_E = 5$, $w_C = 4$, $a_A = a_B = a_D = a_E = 2$, $a_C = 4$.

Let's recall the WAFP gap formulas:

$$g_{LWAFP} = \frac{\sum_{i=1}^{n} \sum_{j \in N(i)} e_{ij} a_j}{\sum_{i=1}^{n} w_i} - \frac{1}{n} \sum_{i=1}^{n} a_i$$

$$g_{SWAFP} = \frac{1}{n} \sum_{i=1}^{n} \left(\frac{1}{w_i} \sum_{j \in N(i)} e_{ij} a_j \right) - \frac{1}{n} \sum_{i=1}^{n} a_i$$

For LWAFP, we get,

$$g_{LWAFP} = \frac{(4 \cdot 2 + 1 \cdot 4) + (4 \cdot 2 + 1 \cdot 4) + (1 \cdot 2 + 1 \cdot 2 + 1 \cdot 2 + 1 \cdot 2) + (4 \cdot 2 + 1 \cdot 4) + (4 \cdot 2 + 1 \cdot 4)}{5 + 5 + 4 + 5 + 5} -$$

$$\frac{1}{5}(2 + 2 + 4 + 2 + 2) = -\frac{1}{15}$$

The sign of this gap should be equal to the sign of $r_{w,a} = r_{w,d}$. The (weighted degree)-degree correlation $r_{w,d}$ is -1, which checks out.

For SWAFP, we get,

$$g_{SWAFP} = \frac{\frac{4\cdot 2+1\cdot 4}{5} + \frac{4\cdot 2+1\cdot 4}{5} + \frac{1\cdot 2+1\cdot 2+1\cdot 2+1\cdot 2}{4} + \frac{4\cdot 2+1\cdot 4}{5} + \frac{4\cdot 2+1\cdot 4}{5}}{5+5+4+5+5} -$$

$$\frac{1}{5}(2+2+4+2+2) = -\frac{23}{12}$$

.

The sign of this gap should be equal to the sign of $r_{\gamma,a} = r_{\gamma,d}$. Recall that $\gamma_i = \sum_{j\in N(i)} \frac{e_{ij}}{w_j}$. So, $\gamma_A = \gamma_B = \gamma_D = \gamma_E = \frac{4}{5} + \frac{1}{4} = \frac{21}{20}$, and $\gamma_C = \frac{1}{5} + \frac{1}{5} + \frac{1}{5} + \frac{1}{5} = \frac{4}{5}$. $r_{\gamma,d}$ is the correlation between $\{\frac{21}{20}, \frac{21}{20}, \frac{4}{5}, \frac{21}{20}, \frac{21}{20}\}$ and $\{2, 2, 4, 2, 2\}$ which is -1, which checks out.

($r_{w,d}$ is equal to $r_{\gamma,d}$ simply because $r_{w,\gamma}$ is equal to 1 for this graph.)

5 If all γ_i are equal in a weighted connected graph, it is a weighted-regular graph

Given a weighted connected graph G on n nodes, where each weight $\in \mathbb{N}^+$, and given a node j of G, we define $N_w(j)$ to be the weighted set of neighbors of j (each neighbor i is present s times where s is the strength of edge (i, j)); we define $w_j = |N_w(j)|$ to be the weighted degree of j; and we define $\gamma_j = \sum_{k\in N_w(j)} \frac{1}{w_k}$. In a non-trivial case of $n > 1$, the weighted set of neighbors of j is non-empty for all j and w_k is positive for all k, so we can expect γ_j to be defined and positive.

Claim: If G is connected and $\gamma_i = \gamma_j$ for all nodes i, j, then $w_i = w_j$ for all nodes i, j.

To prove this claim, we first observe that

$$\sum_{j=1}^{n} \gamma_j = \sum_{j=1}^{n} \sum_{k\in N_w(j)} \frac{1}{w_k}$$

contains the term $\frac{1}{w_k}$ exactly w_k times (since each of k's friends t uses that term in their calculation of γ_x exactly t_x times where t_x is the strength of edge (k, x), and the sum of all such t is equal to w_k), and therefore

$$\sum_{j=1}^{n} \gamma_j = n.$$

It follows that if $\gamma_i = \gamma_j$ for all nodes i, j, then $\gamma_i = 1$ for all nodes i.

Now suppose G contains two nodes whose weighted degrees are not the same. Let a be a node of minimum weighted degree and b be a node of maximum weighted degree; so we have $w_a < w_b$. Since G is connected, there is a path P in G with one end equal to a and the other end equal to b. As we traverse P from a to b, there is a first node j on P for which $w_j \neq w_a$. Node j cannot be the first node on P (since that first node is a with weighted degree w_a and $w_j \neq w_a$), and the node i immediately preceding j on P has $w_i = w_a$, the minimum weighted degree in G (since j is the first node on the path for which its weighted degree is not equal to w_a).

Now observe that $\gamma_i = \sum_{k \in N_w(i)} \frac{1}{w_k}$ is a sum of w_i terms; each of these terms is at most $\frac{1}{w_i}$ since i has the minimum degree in G. Moreover, at least one of these terms is $\frac{1}{w_j}$ since j is a neighbor of i. We know that $\frac{1}{w_j} < \frac{1}{w_i}$. Therefore, since γ_i is a sum of w_i terms, each at most $\frac{1}{w_i}$ and one of the terms strictly less than $\frac{1}{w_i}$, we have $\gamma_i < 1$.

Since $\gamma_i \neq 1$, we conclude that G does not have the property that $\gamma_i = \gamma_j$ for all nodes i, j. That is a contradiction.

It follows that the only connected graphs G with the property that $\gamma_i = \gamma_j$ for all nodes also have the same weighted degree at every node.

For graphs that are not connected, we can apply this argument to each connected component separately, and conclude that all the weighted degrees *in each component* are the same.

6 If all δ_i are equal in an unweighted connected graph, it is a regular graph

This argument is very similar to the argument in the previous section, except that it is applied to an unweighted graph.

Given a connected graph G on n nodes, and given a node j of G, we define $N(j)$ to be the set of neighbors of j; we define $d_j = |N(j)|$ to be the degree of j; and we define $\delta_j = \sum_{k \in N(j)} \frac{1}{d_k}$. In a non-trivial case of $n > 1$, the set of neighbors of j is non-empty for all j and d_k is positive for all k, so we can expect δ_j to be defined and positive.

> *Claim: If G is connected and $\delta_i = \delta_j$ for all nodes i, j, then $d_i = d_j$ for all nodes i, j.*

To prove this claim, we first observe that

$$\sum_{j=1}^{n} \delta_j = \sum_{j=1}^{n} \sum_{k \in N(j)} \frac{1}{d_k}$$

contains the term $\frac{1}{d_k}$ exactly d_k times (once for each of the d_k neighbors of k), and therefore

$$\sum_{j=1}^{n} \delta_j = n.$$

It follows that if $\delta_i = \delta_j$ for all nodes i, j, then $\delta_i = 1$ for all nodes i.

Now suppose G contains two nodes whose degrees are not the same. Let a be a node of minimum degree and b be a node of maximum degree; so we have $d_a < d_b$. Since G is connected, there is a path P in G with one end equal to a and the other end equal to b. As we traverse P from a to b, there is a first node j on P for which $d_j \neq d_a$. Node j cannot be the first node on P (since that first node is a with degree d_a and $d_j \neq d_a$), and the node i immediately preceding j on P has $d_i = d_a$, the minimum degree in G (since j is the first node on the path for which its degree is not equal to d_a).

Now observe that $\delta_i = \sum_{k \in N(i)} \frac{1}{d_k}$ is a sum of d_i terms; each of these terms is at most $\frac{1}{d_i}$ since i has the minimum degree in G. Moreover, at least one of these terms is $\frac{1}{d_j}$ since j is a neighbor of i. We know that $\frac{1}{d_j} < \frac{1}{d_i}$. Therefore, since δ_i is a sum of d_i terms, each at most $\frac{1}{d_i}$ and one of the terms strictly less than $\frac{1}{d_i}$, we have $\delta_i < 1$.

Since $\delta_i \neq 1$, we conclude that G does not have the property that $\delta_i = \delta_j$ for all nodes i, j. That is a contradiction.

It follows that the only connected graphs G with the property that $\delta_i = \delta_j$ for all nodes also have the same degree at every node.

For graphs that are not connected, we can apply this argument to each connected component separately, and conclude that all the node degrees *in each component* are the same.

7 8 gaps for one network: a detailed argument

This is an extended argument that details each of the 8 gaps for the example network from the main text, copied on Figure S4 below:

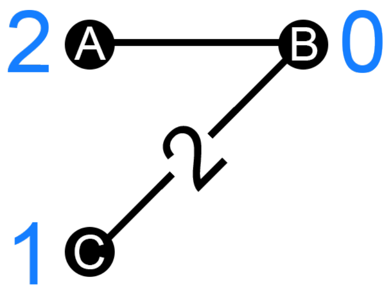

Figure S4: (repeated from the main text) This is a weighted undirected graph G on 3 nodes. The weight of each edge, when not 1, is listed on the edge. The nodes are labeled A through C. Each node has a numeric attribute a_i associated with it, and it's listed in blue next to each node.

Since there are a lot of quantities, let's write the basic ones out: $d_A = 1$, $d_B = 2$, $d_C = 1$,

$w_A = 1$, $w_B = 3$, $w_C = 2$, $a_A = 2$, $a_B = 0$, $a_C = 1$. We will compute deltas and gammas in their associated sections. For better generalizability of the equations, take the number of nodes to be n.

We want to apply an extension (LEFP or SEFP) in every case. In Equations 5 and 6 of the main text, we defined the gaps for LEFP and SEFP via attributes (first-order values) and edge weights (used for second-order calculations):

$$g_{LEFP} = \frac{\sum_{i=1}^{n} \sum_{j \in N(i)} e_{ij} a_j}{\sum_{i=1}^{n} \sum_{j \in N(i)} e_{ij}} - \frac{1}{n} \sum_{i=1}^{n} a_i$$

$$g_{SEFP} = \frac{1}{n} \sum_{i=1}^{n} \left(\frac{1}{\sum_{j \in N(i)} e_{ij}} \sum_{j \in N(i)} e_{ij} a_j \right) - \frac{1}{n} \sum_{i=1}^{n} a_i$$

When disregarding attributes, we should replace a_i with the sum of the weights of the edges that i is part of. That's w_i when considering edge weights and d_i when reducing edge weights to 1. As for $\sum_{j \in N(i)} e_{ij}$, we will replace it with w_i when considering edge weights and with d_i when reducing edge weights to 1 as well.

7.1 Friendship Paradox

To look at the two versions of the basic Friendship Paradox, we need a simple (unweighted) graph. To that end, we "simplify" the edge BC to have weight 1. We also take the node attributes to be equal to weighted degrees, which are equal to degrees once we have disregarded edge weights. To make it clearer just what version we are looking at, we will actually denote our first-order values d_i and not w_i or a_i.

The list FP gap is equal to the mean second-order attribute (list version) minus the mean attribute in G, or the mean second-order degree (list version) minus the mean degree in G. The list second-order degree of each node is simply the list of its friends' degrees, so for node B, it's $[d_A, d_C]$. The length of this list is equal to d_B. The mean second-order degree (list version)

is the mean of the concatenation of all such lists across all the nodes, which is expressed as the sum of the elements across all the lists divided by the total length of the lists. The total length of the lists is the sum of all node degrees.

The LFP gap for G is expressed as:

$$g_{LFP} = \frac{(d_B) + (d_A + d_C) + (d_B)}{d_A + d_B + d_C} - \frac{d_A + d_B + d_C}{n} =$$

$$\frac{(2) + (1+1) + (2)}{1+2+1} - \frac{1+2+1}{3} = \frac{1}{6} > 0$$

LFP holds.

The singular FP gap is equal to the mean second-order degree (singular version) minus the mean degree in G. The singular second-order degree of each node is the mean of its friends' degrees, so for node B, it's $\frac{d_A+d_C}{2}$. The mean second-order degree (singular version) is the mean of the singular second-order degrees across all the nodes.

The SFP gap for G is expressed as:

$$g_{SFP} = \frac{\frac{d_B}{d_A} + \frac{d_A + d_C}{d_B} + \frac{d_B}{d_C}}{n} - \frac{d_A + d_B + d_C}{n} =$$

$$\frac{\frac{2}{1} + \frac{1+1}{2} + \frac{2}{1}}{3} - \frac{1+2+1}{3} = \frac{1}{3} > 0$$

SFP holds.

7.2 Attribute-based Friendship Paradox

AFP deals with simple graphs that have node attributes. Here, we disregard the edge weights but do use the node attributes. This means that for our extension formula, each edge weight is equal to 1 and each attribute is equal to the number in blue.

The list AFP gap is equal to the mean second-order attribute (list version) minus the mean attribute in G. The list second-order attribute of each node is the list of its friends' attributes,

so for node B, it's $[a_A, a_C]$. The length of this list is d_B. Remember that we are not using edge weights here so each friend is represented once. The mean second-order attribute (list version) is the mean of the concatenation of all such lists across all the nodes.

The LAFP gap for G is expressed as:

$$g_{LAFP} = \frac{(a_B) + (a_A + a_C) + (a_B)}{d_A + d_B + d_C} - \frac{a_A + a_B + a_C}{n} =$$
$$\frac{(0) + (2+1) + (0)}{1+2+1} - \frac{2+0+1}{3} = -\frac{1}{4} < 0$$

LAFP fails.

We saw in Section 4.1.2 of the main text that the sign of g_{LEFP} is equal to the sign of the (weighted degree)-attribute correlation $r_{w,a}$ when the latter is defined. In this case of no edge weights, the weighted degree sequence is equal to the degree sequence, and $r_{w,a} = r_{d,a}$. The correlation between $\{1, 2, 1\}$ and $\{2, 0, 1\}$, $r_{d,a}$, is equal to -0.866, which checks out.

The singular AFP gap is equal to the mean second-order attribute (singular version) minus the mean attribute in G. The singular second-order attribute of each node is the mean of its friends' attributes, so for node B, it's $\frac{a_A + a_C}{2}$. The mean second-order attribute (singular version) is the average across such values for all the nodes.

The SAFP gap for G is expressed as:

$$g_{SAFP} = \frac{\frac{a_B}{d_A} + \frac{a_A + a_C}{d_B} + \frac{a_B}{d_C}}{n} - \frac{a_A + a_B + a_C}{n} =$$
$$\frac{\frac{0}{1} + \frac{2+1}{2} + \frac{0}{1}}{3} - \frac{2+0+1}{3} = -\frac{1}{2} < 0$$

SAFP fails.

We saw in Section 4.2.2 of the main text that the sign of g_{SEFP} is equal to the sign of the gamma-attribute correlation $r_{\gamma,a}$ when the latter is defined. In this case of no edge weights, the weighted degree sequence is equal to the degree sequence, the gamma sequence is equal to the

delta sequence, and $r_{\gamma,a} = r_{\delta,a}$. We can check that $r_{\delta,a}$ is indeed negative given the negative gap sign. Delta of a node is equal to the sum of reciprocal degrees of its friends: $\delta_i = \sum_{j \in N(i)} \frac{1}{d_j}$. So, $\delta_A = \frac{1}{d_B} = \frac{1}{2}$, $\delta_B = \frac{1}{d_A} + \frac{1}{d_C} = \frac{1}{1} + \frac{1}{1} = 2$, and $\delta_C = \frac{1}{d_B} = \frac{1}{2}$. The correlation between $\{\frac{1}{2}, 2, \frac{1}{2}\}$ and $\{2, 0, 1\}$, $r_{\delta,a}$, is equal to -0.866, which checks out.

($r_{d,a} = r_{\delta,a}$ because $r_{d,\delta}$ is equal to 1 for this graph.)

7.3 Weighted Friendship Paradox

For the two versions of WFP, we consider the edge weights and disregard the node attributes. This means that the attributes are equal to the weighted degrees, and we denote them w_i.

The list WFP gap is equal to the mean second-order attribute (list version, taking weights into account) minus the mean attribute in G, or the mean second-order weighted degree (list version) minus the mean weighted degree in G. As a reminder, the weighted degree w_i of node i is the sum of weights of all edges that i is part of. The list second-order weighted degree of each node is the list of its friends' weighted degrees, **weighted by** the strength of the node's edges with each of the friends. So for node B, it's $[w_A, w_C, w_C]$, since the edge AB has weight 1 and the edge BC has weight 2. The length of such a list is w_B, and the length of the concatenation of all such lists across the nodes is the sum of all weighted degrees. The mean of this big list is the mean second-order weighted degree (list version), expressed as the sum of all the list elements divided by its length.

The LWFP gap for G is expressed as:

$$g_{LWFP} = \frac{(w_B) + (w_A + w_C + w_C) + (w_B + w_B)}{w_A + w_B + w_C} - \frac{w_A + w_B + w_C}{n} =$$

$$\frac{(3) + (1 + 2 + 2) + (3 + 3)}{1 + 3 + 2} - \frac{1 + 3 + 2}{3} = \frac{1}{3} > 0$$

LWFP holds.

The singular WFP gap is equal to the mean second-order attribute (singular version, taking weights into account) minus the mean attribute in G, or to the mean second-order weighted degree (singular version) minus the mean weighted degree in G. The singular second-order weighted degree of each node is the mean of the list second-order weighted degree of the node, so for node B, it would be the mean of the list $[w_A, w_C, w_C]$, or $\frac{w_A+w_C+w_C}{3}$, 3 being w_B. The mean second-order weighted degree (singular version) is the average across such values for all the nodes.

The SWFP gap for G is expressed as:

$$g_{SWFP} = \frac{\frac{w_B}{w_A} + \frac{w_A + w_C + w_C}{w_B} + \frac{w_B + w_B}{w_C}}{n} - \frac{w_A + w_B + w_C}{n} =$$

$$\frac{\frac{3}{1} + \frac{1+2+2}{3} + \frac{3+3}{2}}{3} - \frac{1+3+2}{3} = \frac{5}{9} > 0$$

SWFP holds.

7.4 Weighted Attribute-based Friendship Paradox (EFP)

Finally, for the two versions of WAFP (EFP) we consider both the edge weights and the node attributes.

The list WAFP gap is equal to the mean second-order attribute (list version, taking edge weights into account) minus the mean attribute in a graph. Here, the node-specific calculations are the same as in the case of LWFP, except that we look at attributes instead of weighted degrees as first-order values. In particular, the list second-order attribute of each node is the list of its friends' attributes, weighted by the strength of the node's edges with each of the friends. So for node B, it's $[a_A, a_C, a_C]$. The length of this list is w_B.

The LWAFP gap for G is expressed as:

$$g_{LWAFP} = \frac{(a_B) + (a_A + a_C + a_C) + (a_B + a_B)}{w_A + w_B + w_C} - \frac{a_A + a_B + a_C}{n} =$$

$$\frac{(0)+(2+1+1)+(0+0)}{1+3+2} - \frac{2+0+1}{3} = -\frac{1}{3} < 0$$

LWAFP fails.

We saw in Section 4.1.2 of the main text that the sign of g_{LWAFP} is equal to the sign of the (weighted degree)-attribute correlation $r_{w,a}$ when the latter is defined. The correlation between $\{1,3,2\}$ and $\{2,0,1\}$, $r_{w,a}$, is equal to -1, which checks out.

The singular WAFP gap is equal to the mean second-order attribute (singular version, taking edge weights into account) minus the mean attribute in a graph. The singular second-order attribute of each node is the mean of the list second-order attribute of the node, so for node B, it would be the mean of the list $[a_A, a_C, a_C]$, or $\frac{a_A+a_C+a_C}{3}$, where 3 is w_B. We take a mean of these means to get the mean second-order attribute (singular version).

The SWAFP gap for G is expressed as:

$$g_{SWAFP} = \frac{\frac{a_B}{w_A} + \frac{a_A+a_C+a_C}{w_B} + \frac{a_B+a_B}{w_C}}{n} - \frac{a_A+a_B+a_C}{n} =$$

$$\frac{\frac{0}{1} + \frac{2+1+1}{3} + \frac{0+0}{2}}{3} - \frac{2+0+1}{3} = -\frac{5}{9} < 0$$

SWAFP fails.

We saw in Section 4.2.2 of the main text that the sign of g_{SWAFP} is equal to the sign of the gamma-attribute correlation $r_{\gamma,a}$ when the latter is defined. We can check that $r_{\gamma,a}$ is indeed negative given the negative gap sign. Gamma of a node is equal to the **weighted** sum of reciprocal weighted degrees of its friends: $\gamma_i = \sum_{j \in N(i)} \frac{e_{ij}}{w_j}$. So, $\gamma_A = \frac{e_{AB}}{w_B} = \frac{1}{3}$, $\gamma_B = \frac{e_{BA}}{w_A} + \frac{e_{BC}}{w_C} = \frac{1}{1} + \frac{2}{2} = 2$, and $\gamma_C = \frac{e_{CB}}{w_B} = \frac{2}{3}$. The correlation between $\{\frac{1}{3}, 2, \frac{2}{3}\}$ and $\{2,0,1\}$, $r_{\gamma,a}$, is equal to -0.944, which checks out.

8 Standard deviations for the correlations in the simulation

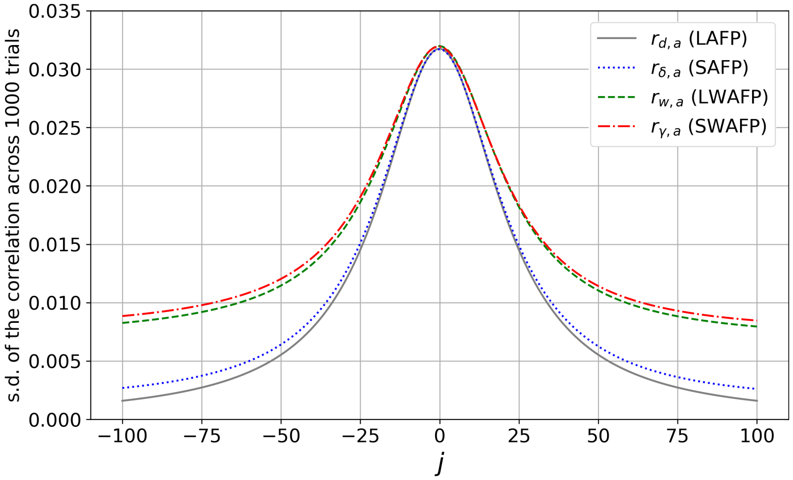

Figure S5: Each of the conditions has 1000 networks, each with an attribute sequence that depends on the degree sequence. For each condition and each correlation type (see legend), we reported the mean of the 1000 correlations in the main text. Here we show the standard deviation of these 1000 correlations. The standard deviation is highest at $j = 0$ where the attributes are independent of the degrees and are simply drawn from the standard normal distribution. As j grows in absolute value, each attribute is affected by adding a fraction of its node's degree to it. As a result, the randomness of the initial attribute sequence matters less and the standard deviation drops.

9 An image of the altered example network discussed in the Data section of the main text

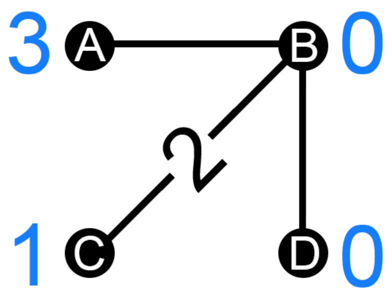

Figure S6: We retain the non-1 edge weight for edge (B, C) here so that the only alterations to the example network from the main text are changing the attribute of node A and adding node D as mentioned. Since we are looking at LAFP, though, we disregard the edge weights in our gap calculations.

10 Plots for the Facebook100 configuration model results

x-axis: original data; y-axis: configuration model.

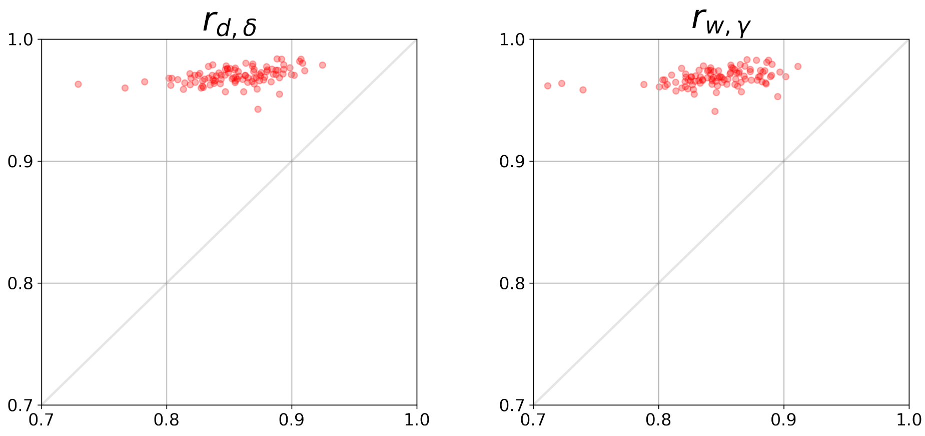

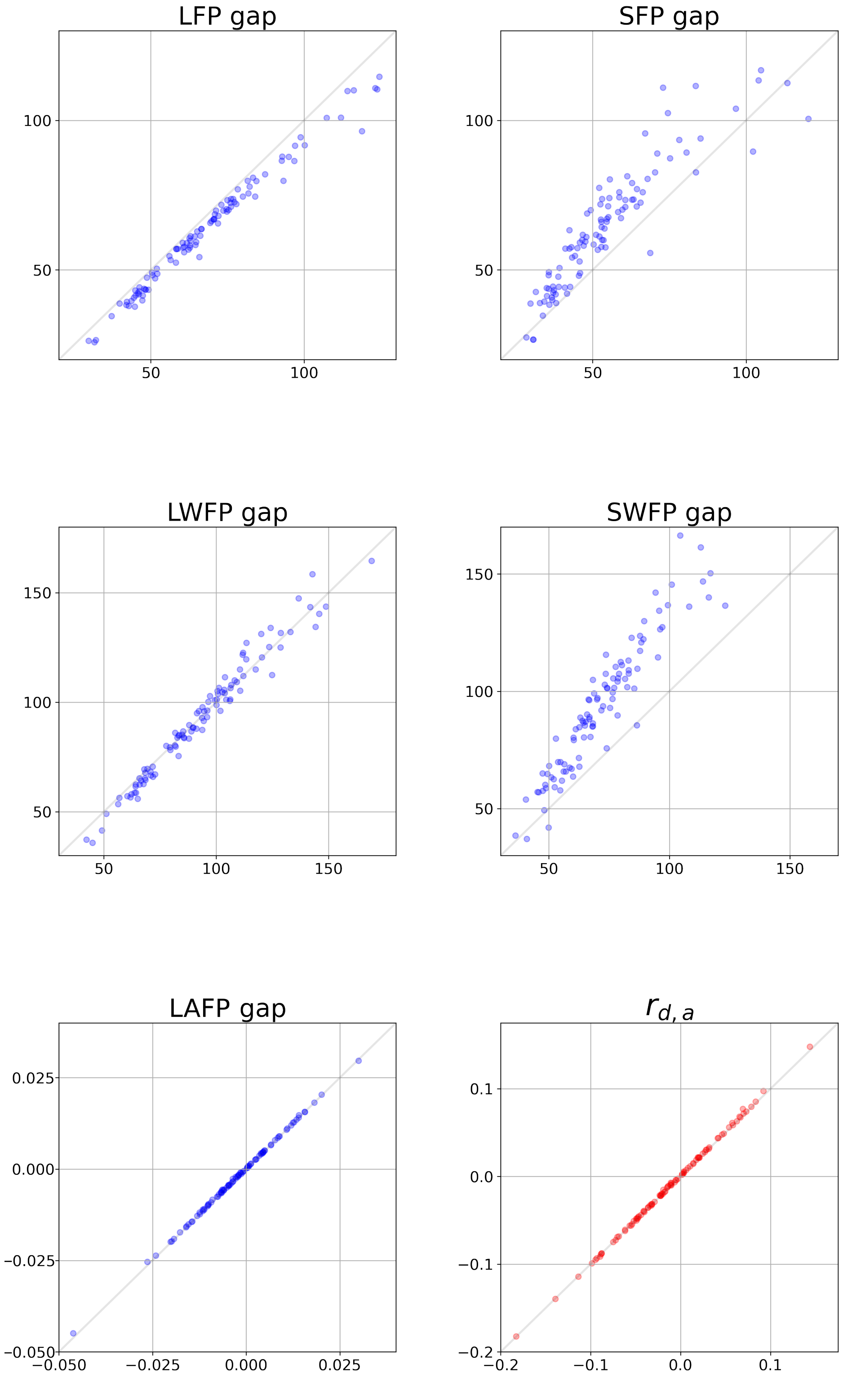
LFP gap
50
100
SFP gap
50
100
LWFP gap
50
100
150
SWFP gap
50
100
150
LAFP gap
−0.050
−0.025
0.000
0.025
$r_{d,a}$
−0.2
−0.1
0.0
0.1

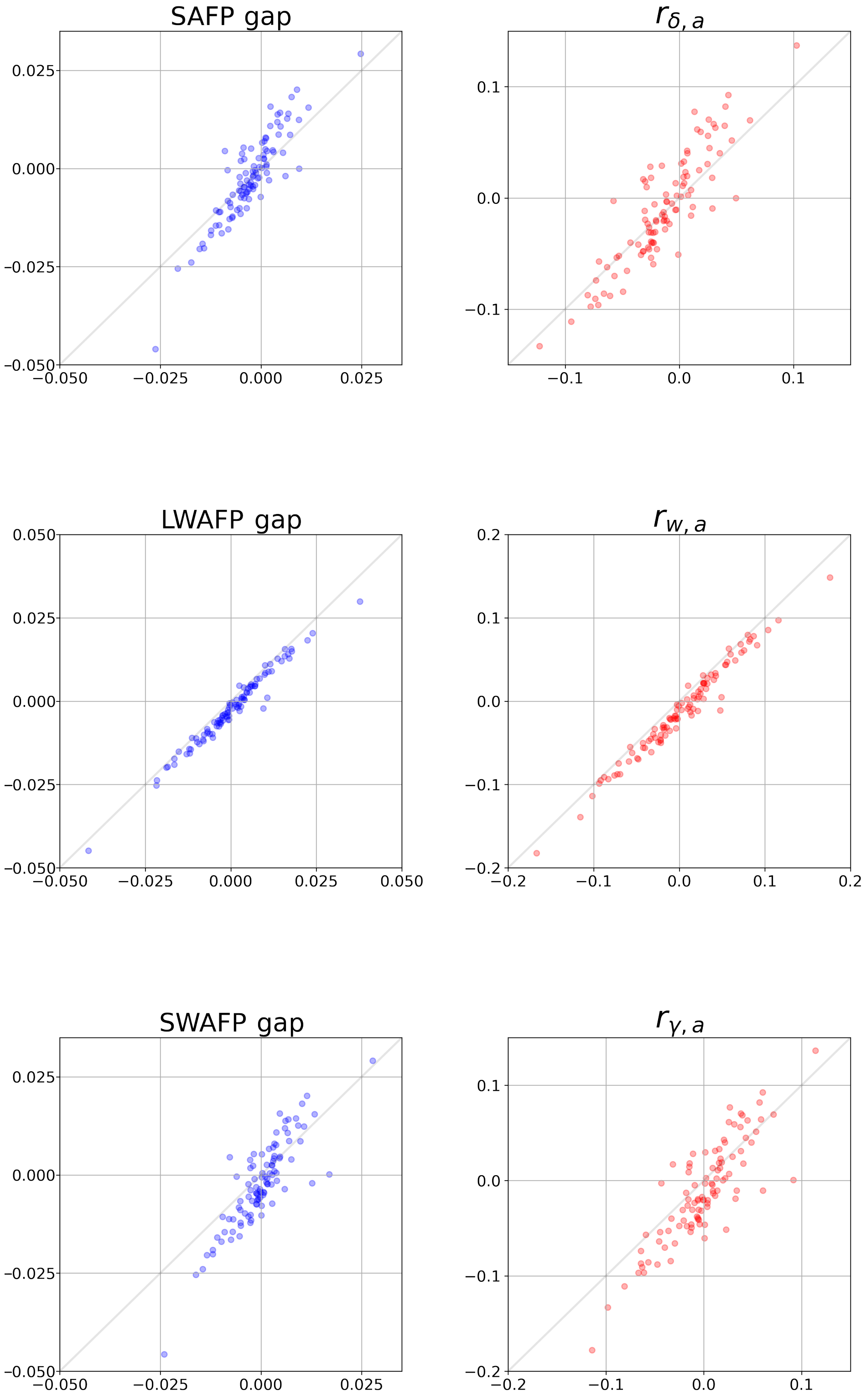
SAFP gap
0.025
0.000
−0.025
−0.050
−0.050
−0.025
0.000
0.025
$r_{\delta,a}$
0.1
0.0
−0.1
−0.1
0.0
0.1
LWAFP gap
0.050
0.025
0.000
−0.025
−0.050
−0.050
−0.025
0.000
0.025
0.050
$r_{w,a}$
0.2
0.1
0.0
−0.1
−0.2
−0.2
−0.1
0.0
0.1
0.2
SWAFP gap
0.025
0.000
−0.025
−0.050
−0.050
−0.025
0.000
0.025
$r_{\gamma,a}$
0.1
0.0
−0.1
−0.2
−0.2
−0.1
0.0
0.1
